# Continuous β^−^ particle exposure: A study of DNA damage in *ex vivo* peripheral blood mononuclear cells irradiation with Radioiodine

**DOI:** 10.1016/j.ctro.2025.101040

**Published:** 2025-09-01

**Authors:** Laura Mazzitelli-Fuentes, Lara Negrin, Virginia Venier, Humberto Romano, Lucia Pereira, Jerónimo Leberle, Maria Soledad Ausas, Ananya Choudhury, Luisa V. Biolatti

**Affiliations:** a National Atomic Energy Commission (CNEA), San Carlos de Bariloche, Argentina; b Institute of Nuclear Technologies for Health Foundation (INTECNUS), San Carlos de Bariloche, Argentina; c National Scientific and Technical Council (CONICET), Argentina; d Division of Cancer Sciences, University of Manchester, UK

**Keywords:** Radiobiology, DNA damage, Targeted radionuclide therapy, Low dose rate, γ-H2AX foci, Chromosomal aberrations

## Abstract

**Background and purpose:**

Targeted radionuclide therapy (TRT) is a promising cancer treatment, but insufficient knowledge of the biological dose–response relationships limits its efficacy. The aim of this study was to characterize DNA damage in peripheral blood mononuclear cells (PBMCs) exposed to β-continuous irradiation (^131^I), and to assess its relationship with the absorbed dose, using two biomarkers: dicentric chromosomes and γ-H2AX foci.

**Materials and methods:**

PBMCs from healthy donors were exposed to ^131^I at various activities (0.37–3.7 MBq) and times (1, 4, 24 h). Absorbed doses were calculated using the Medical Internal Radiation Dose formalism. DNA damage was assessed by chromosomal aberration frequency and γ-H2AX foci quantification. Lithium chloride (LiCl) was used to evaluate the rescue of delayed foci formation after 24 h exposure.

**Results:**

Continuous β-irradiation induced a linear increase in CAs (α = 0.0643 ± 0.0068), in contrast to the linear-quadratic response observed in acute X-ray exposure (α = 0.0359 ± 0.0093, β = 0.0673 ± 0.0042). The frequency of CAs is lower under radionuclide irradiation compared to X-rays. γ-H2AX increased significantly at 1 and 4 h of continuous exposure but diminished at 24 h, despite continuous irradiation. LiCl treatment partially restored γ-H2AX foci levels at 24 h.

**Conclusion:**

Dose-response relationship under continuous β-irradiation, assessed by CAs, follows a linear trend. DNA damage induced foci show a time-dependent dynamic. The decline in foci at 24 h, reversible with LiCl, highlights the limitations of γ-H2AX as a biomarker under prolonged irradiation conditions. These findings emphasize the need for optimized dosimetry methods and identify reliable biomarkers in TRT.

Abbreviations: TRT, targeted radionuclide therapy; MIRD, Medical Internal Radiation Dose; EBRT, external beam radiotherapy; LQ, linear-quadratic; DCA, dicentric chromosome assay; DSB, double strand break; PBMCs, peripheral blood mononuclear cells; PB, peripheral blood; FBS, fetal bovine serum; PBS, phosphate-buffered saline; CAs, chromosomal aberrations; RT, room temperature.

## Highlights

• Continuous low-dose radiation causes different biological effects than acute exposure.
• DNA damage from continuous exposure follows a linear, not a linear-quadratic, model.
• Chromosomal damage from ^131^I is lower than from X-rays at the same dose.
• γH2AX foci increase initially but decline after 24 h of continuous exposure.
• Lithium chloride restores γH2AX foci, revealing hidden damage during prolonged exposure.

## Graphical abstract

**Figure:**
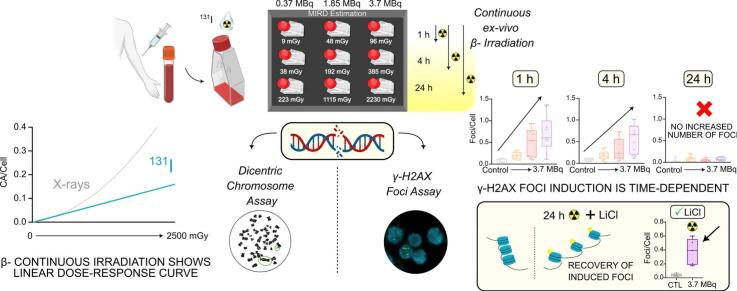

## Introduction

In the last few decades, Targeted Radionuclide Therapy (TRT) has emerged as a promising therapeutic strategy for targeting and eradicating cancer cells [[1], [2], [3]]. Although this approach is highly specific and minimally invasive, its main limitation is the lack of studies on the analysis and correlation between dose rate, absorbed doses, and their biological and clinical effects. The tumor response, the fraction of surviving cells, and toxicity in healthy organs will primarily depend on the amount of absorbed energy in the exposed regions, i.e., the absorbed dose.

Fixed standardized activities are administered in routine clinical practice according to international procedure guidelines, but this results in very different absorbed doses among patients [[4], [5], [6], [7], [8], [9], [10], [11]]. Absorbed doses are not routinely calculated because of the complexity and time-consuming methodologies of dose calculation (e.g., multiple imaging requirements, complex computational models) and the lack of information on dose-biological effect correlations [12,13]. Insufficient radiobiologic data in TRT leads to the extrapolation of findings from external beam radiotherapy (EBRT) to predict dose–response relationship derived from models, such as the linear-quadratic (LQ) model [14]. However, the irradiation patterns between TRT and EBRT are markedly different. TRT delivers a continuous and decreasing dose rate in a heterogeneous exposure, while in EBRT, the irradiation is homogeneous, acute, and at a high-dose rate [15]. Biological effects differ between both therapies, which highlights the need of studying the radiobiological aspects associated with TRT [[16], [17], [18]].

Biodosimetry refers to the detection of radiation-induced biological alterations which allows estimation of absorbed doses through methodologies assessing effects such as DNA damage, in particular double-strand breaks (DSBs). In TRT, biodosimetry provides valuable dosimetric information that could be used alongside image-based assessments or independently, especially in cases where image-based dosimetry is complex.

The dicentric chromosome assay (DCA), constitutes the gold standard technique in external biodosimetry and is a widely and medically recognized method [19]. During the repair of DSBs caused by ionizing radiation, improper repair of broken chromosomes and abnormal chromosome replication can lead to the formation of dicentric chromosomes. The frequency of dicentrics follows the linear-quadratic (LQ) model curve and can be used to estimate the absorbed dose. This model describes radiation-induced cellular responses through two coefficients: the linear coefficient α, attributed to damage from a single electron track producing DSB, related to irreparable lethal damage, and the quadratic coefficient β, resulting from two independent tracks, each generating a single break in separate strands, associated with sublethal damage.

The γH2AX foci assay, which consists in identifying the sites of DSBs of DNA by labelling with phosphorylated histone H2AX, can investigate the time- and dose-dependency of DNA DSBs induction and repair [20,21]. This technique was proven to be very sensitive in terms of detecting discrete nuclear foci at DSB sites, even at low acute dose exposures [22,23]. In contrast with cytogenetic assays, this method is particularly advantageous since it does not require cell culturing and allows for the analysis of large sample sizes.

In TRT, DCA allows cumulative dose estimations; however, it is strongly influenced by the dose rate, which should be taken into account to avoid underestimation of the absorbed dose [24]. Regarding the γH2AX assay, *in vitro* studies show a rapid increase in the number of foci after exposure, followed by an exponential decrease within 24 h due to DNA repair [25]. Most of these studies have been conducted under experimental conditions of acute radionuclide irradiation, not addressing the continuous irradiation conditions, as it occurs during clinical practice.

We evaluated the *ex vivo* radiation-induced effect in PBMCs exposed to continuous irradiation, at different time points with ^131^I. The aim of this study was to characterize DNA damage response under continuous irradiation conditions with a β^−^ emitter using established biomarkers. This was achieved through an experimental framework designed to simulate the impact of dose rate variations during continuous irradiation. To our knowledge, this is the first study to investigate DSBs under protracted irradiation with a radionuclide over long time periods in an *ex vivo* setting.

## Materials and methods

**Blood Sampling, Cell Culture, and Irradiation.** Peripheral blood (PB) was obtained from healthy volunteers (4 men, 9 women; mean age ± SD: 36.9 ± 3.9). Blood samples from each donor was drawn into Li-heparin tubes, diluted 1:1 with RPMI-1640 medium (pH = 7.2) supplemented with 10 % Fetal Bovine Serum (FBS) and 1 % Penicillin-Streptomycin (10.000 U/ml), and incubated with 0.37, 1.85 or 3.7 MBq of [^131^I]NaI solution (Radiofarma, Bacon) at 37 °C for 1, 4 and 24 h. Additionally, for comparison with external-beam irradiation, PB samples from two healthy donors (1 man and 1 woman: 30 and 35 years old, respectively) were irradiated with 15 MV X-rays at doses of 0.10, 0.25, 0.5, 1.0, 1.5, 2.0, 3.0 y 4.0 Gy using a clinical linear accelerator (Elekta Synergy® Platform). After centrifugation, the supernatant was discarded and pellet washed with RPMI-1640. A cell viability assay was performed using the Live/Dead Cell Viability/Cytotoxicity Assay Kit (30002, Biotium, Fremont, CA) to confirm that there were no significant cytotoxic effects under the culture conditions (see Supplementary Material A). Similar viability was observed across all treatments (see Supplementary Material B, Supp. Fig. 1).

**Fig. 1:**
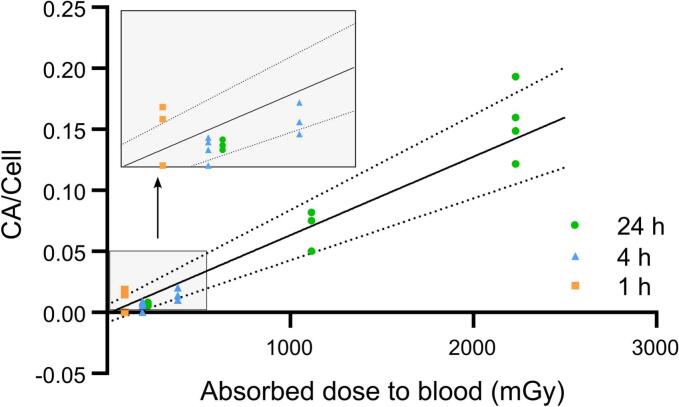
**Dose-Response Curve of Chromosomal Aberrations Induced by ^131^I in Peripheral Lymphocytes.** Dose-response curve obtained from peripheral lymphocytes from healthy volunteers exposed to different ^131^I activities (0.37 MBq, 1.85 MBq, 3.7 MBq) for 1, 4 and 24 h. Each data point per condition represents an individual, n = 4. Solid line depicts the following simple linear model fit: Y = 0.0643(± 0.0068) D − 0.0011 (± 0.0035), (χ^2^_(16)_ = 20.08, p = 0.902). Dotted lines show 95 % confidence intervals. Inset shows a zoomed view of the axis, determining the minimal detection of CA at 0.1 Gy. CA, chromosomal aberrations.

**Calculation of Absorbed Dose to Blood.** Absorbed doses were calculated using the MIRD method [26]. In our *ex vivo* model, blood is both the target and the source organ. The absorbed dose was determined by:

(1) $$D_{\mathrm{blood}}=\tilde{A}S\left(r_{\mathrm{blood}}\leftarrow r_{\mathrm{blood}}\right)$$

where the cumulated activity (Ã), or integral of activity over time, was calculated as:

(2) $$\tilde{A}=\frac{A_{0}}{\lambda_{\mathrm{eff}}}\left(1-e^{-\lambda t}\right)$$

Since biological decay is absent, physical decay equals effective decay, $\lambda_{\mathrm{eff}}=\lambda_{p}$.

The S factor was calculated as follows:

(3) $$S\left(r_{\mathrm{blood}}\leftarrow r_{\mathrm{blood}}\right)=\sum_{i}^{\;}\frac{y_{i}E_{i}\phi_{i}}{m_{T}},$$

where *m_T_* = 0.004 kg, and φ = 1.

A homogeneous distribution of the radioisotope was assumed (4 ml liquid solution, *δ* = 1 g/ml).

**Dicentric Chromosome Assay.** DCA was performed using the IAEA’s Cytogenetic Dosimetry Guideline [19]. Metaphases were acquired and analyzed using the CytoVision® Image Analysis and Capture System (Leica Microsystems). A minimum of 600 metaphases were scored for each treatment. Dose-response curves were adjusted to the linear or linear-quadratic model using the iterative weighted least squares method in Dose Estimate_v5.3 software [27].

**Immunofluorescence and Image Acquisition.** DSBs were detected using the γ-H2AX foci assay. After irradiation, PBMCs were isolated by Ficoll-Paque PLUS (Cytiva) gradient centrifugation, washed, and fixed in 4 % PFA. Cells were blocked for 1 h and incubated overnight at 4 °C with primary antibodies (rabbit polyclonal anti-Phospho-Histone H2A.X (Ser139) 1:800 (2577, Cell Signal)), and then with Alexa Fluor 488 (4412, Cell Signal) secondary antibody (1:1000) for 2 h at RT. Samples were mounted in glycerol, and images acquired on a Confocal Fluorescence Microscope (LSM 980, Carl Zeiss) and foci quantification was performed with FIJI software (ImageJ v1.53).

**Lithium Chloride Treatment.** To investigate whether foci induction is influenced by chromatin state, LiCl (3019741, Thermofisher) was added to control, 0.37 or 3.7 MBq cell cultures at a final concentration of 20 mM, according to Oizumi et al. 2024 [28], for 4 and 24 h at 37 °C. The procedures described previously were followed.

**Statistical Analysis.** Data were analyzed using GraphPad Prism version 8.0.1 (GraphPad Software, CA, USA). Every data set was tested by the Grubb’s outlier test, with alpha = 0.05. Normality was assessed using the Shapiro-Wilk test. Homoscedasticity was analyzed by Bartlett's test. In scatter plots, data points are shown as mean ± SEM. Box plots display the distribution of the data, with the box representing the interquartile range (IQR), line indicates median, and whiskers extending to the minimum and maximum values. Statistical significance was assumed when p < 0.05. For multiple comparisons, Dunn’s test was applied. Statistical significance is indicated as follows: *p < 0.05; **p < 0.01; ***p < 0.001; ****p < 0.0001. The equations resulting from fits have been added to Supplementary Material B.

Detailed description of methods is included in “Supplementary Material A”.

## Results

**Absorbed Dose to Blood.** MIRD estimated absorbed doses in cell cultures under continuous irradiation with ^131^I ranged from 9-2230 mGy. Table 1 summarizes the absorbed doses for each time and administered activity. After 24 h of cell culture, which was the maximum duration of our assays, the absorbed dose rate of the radionuclide decreased by 8.28 % compared to the initial measurement (Supp. Fig. 2).

**Table 1:** Absorbed Doses to Blood for Each Time and Administered Activity, Calculated by MIRD Method.

	**0.37 MBq**	**1.85 MBq**	**3.7 MBq**
**1 h**	*9 mGy*	*48 mGy*	*96 mGy*
**4 h**	*38 mGy*	*192 mGy*	*385 mGy*
**24 h**	*223 mGy*	*1115 mGy*	*2230 mGy*

**Fig. 2:**
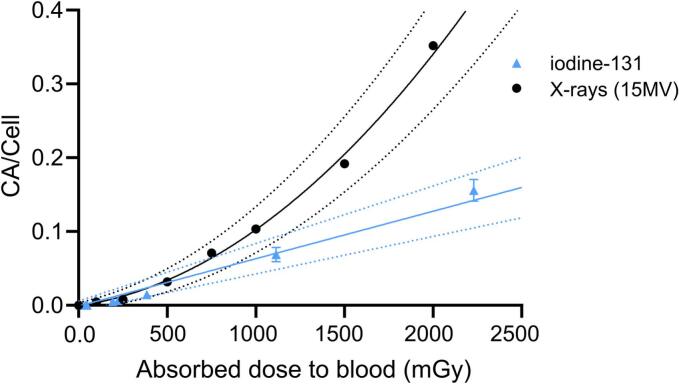
**Dose-Response Curve of Chromosomal Aberrations Induced by ^131^I and X-rays in Peripheral Lymphocytes**. Dose-response curve obtained of peripheral lymphocytes from healthy volunteers exposed to different ^131^I activities for 1, 4 and 24 h and X-rays of 15 MV. Solid line with circular markers depicts linear-quadratic model fit in X-ray: Y = 0.0673 (± 0.0042) D^2^ + 0.0359 (± 0.0093) D − 0.0007 (± 0.0021), (χ^2^_(11)_ = 12.78, p = 0.308). Solid line with triangular markers depicts the following simple linear model fit ^131^I: Y = 0.0643 (± 0.0068) D − 0.0011 (± 0.0035), (χ^2^_(16)_ = 20.08, p = 0.902). Dashed lines show 95 % confidence intervals. CA, chromosomal aberrations. n = 4 for ^131^I; n = 2 for X-rays.

**Dose-Response Curve of Chromosomal Aberrations Induced by ^131^I and X-rays in Peripheral Lymphocytes.** CAs were quantified for each condition in peripheral lymphocytes. A linear and statistically significant increase in CAs was observed at absorbed doses above 96 mGy across all analyzed times: 1, 4 and 24 h. The CA/cell ratios observed were 0.0063 for 96 mGy over 1 h; 0.005 and 0.014 for 192 and 385 mGy, respectively in 4 h; 0.006, 0.07 and 0.15 for 223, 1115 and 2230 mGy in 24 h (Fig. 1).

A comparative analysis of CAs frequencies induced by X-ray and ^131^I radiation was conducted. A consistent frequency of CAs was detected at doses below ∼ 500 mGy, following a similar trend to that observed in acute X-ray exposure (Fig. 2). At higher absorbed doses, achieved at 24 h of continuous irradiation with the β^−^ emitter, the frequency of CAs per dose was significantly lower than its equivalent for X-rays. Continuous exposure to ^131^I produced a linear dose–response relationship, described by the equation Y = 0.0643(± 0.0068) D **−** 0.0011 (± 0.0035). In contrast, X-rays exposure followed a linear-quadratic relationship (Y = 0.0673 (± 0.0042) D^2^ + 0.0359 (± 0.0093) D **−** 0.0007 (± 0.0021)). This contrast underscores the minimal influence of the quadratic coefficient of LQ model during continuous irradiation.

**Fig. 3:**
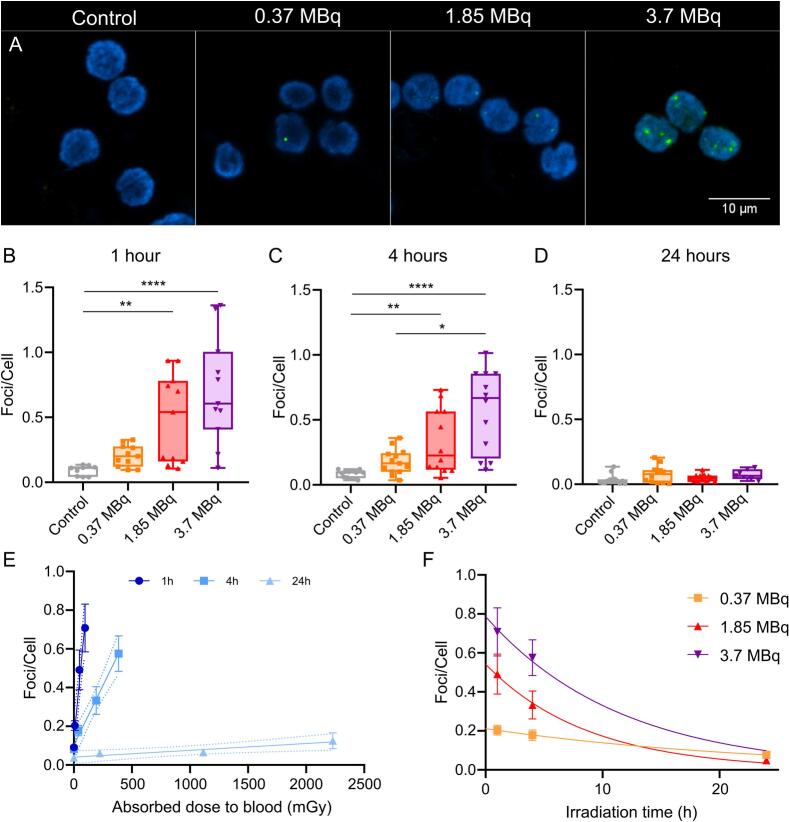
**γ-H2AX Foci Induction by ^131^I in Peripheral Blood Mononuclear Cells is Time-Dependent. A.** Representative image of γ-H2AX foci induced *ex vivo* in PBMCs from healthy individuals exposed to ^131^I for 1 h. Indirect immunofluorescence for γ-H2AX (green) and nuclear counterstain with Hoechst 33,258 (blue). **B-D.***Ex vivo* measurement of γ-H2AX foci in PBMCs from healthy individuals exposed to different activities of ^131^I for 1 h (**B**. Kruskal-Wallis test, K-W = 20.98, p < 0,0001, Control, n = 9; 0.37–3.7 MBq, n = 11), 4 h (**C.** Kruskal-Wallis test, K-W = 26.02, p < 0,0001, Control, n = 13; 0.37 MBq, n = 13; 1.85 MBq, n = 12; 3.7 MBq, n = 12**)**, and 24 h (**D.** Kruskal-Wallis test, K-W = 7.273, n.s. Control, n = 13; 0.37 MBq, n = 12; 1.85 MBq, n = 12; 3.7 MBq, n = 7). **E.***Ex vivo* measurement of γ-H2AX foci in PBMCs from healthy individuals exposed to ^131^I for 1, 4, and 24 h as a function of absorbed dose (Gy). Each data point represents the mean of independent experiments. Solid lines depict linear regressions. Dashed lines show 95 % confidence intervals. Differences between slopes are significant (ANOVA, F_(2, 130)_ = 50.86, p < 0.0001). **F.***Ex vivo* measurement of γ-H2AX foci in PBMCs from healthy individuals exposed to three different activities of ^131^I as a function of time. Solid lines depict exponential fit. Models are significantly different (ANOVA, F_(4, 95)_ = 11.62, p < 0.0001). PBMCs, peripheral blood mononuclear cells. (For interpretation of the references to colour in this figure legend, the reader is referred to the web version of this article.)

**Time-Dependent Induction of γ-H2AX Foci by ^131^I in Peripheral Blood Mononuclear Cells.** To study the DNA damage produced at different activities and times under β^−^ continuous irradiation, the induction of DSBs was analyzed by detecting γ-H2AX foci (Fig. 3A). An increase in the average number of γ-H2AX foci per cell was observed in the samples irradiated for 1 and 4 h compared to unexposed controls (Fig. 3, B-C). At these time points, the increase in foci correlated positively with the activity administered to the culture. The mean number of foci per cell was 0.09100 ± 0.01304 (control), 0.2039 ± 0.02530 (0.37 MBq), 0.4912 ± 0.1027 (1.85 MBq) and 0.7077 ± 0.1237 (3.7 MBq) after 1 h, and 0.08232 ± 0.00817 (control), 0.1780 ± 0.02679 (0.37 MBq), 0.3325 ± 0.07181 (1.85 MBq) and 0.5754 ± 0.09137 (3.7 MBq) after 4 h. At 24 h of culture, no radiation-induced foci were observed in any treatment, not even in the culture with 3.7 MBq, despite its high dose rate (Fig. 3D). This contrasts with the induction of foci in cultures exposed to radionuclides at earlier time points. The analysis of focus induction as a function of absorbed dose (Fig. 3E) shows an increased frequency of foci at 1 and 4 h compared to 24 h, despite higher doses reached at longer times. In summary, under these experimental conditions, foci induction under continuous irradiation is time-dependent and diminishes with prolonged exposure (Fig. 3F).

To rule out the possibility that the observed decrease in foci is associated with culture conditions, PB cultures were maintained for 24 h, and then irradiated for 1 h with 3.7 MBq of ^131^I (Supp. Fig. 3). The radiation-induced foci levels were comparable to those observed in cells irradiated immediately after culturing (Supp.Fig. 3 A-D). We investigated whether any factor in the irradiated culture supernatant after 24 h could inhibit foci induction. The supernatant from a 24-h culture exposed to 3.7 MBq was added to a fresh PB culture for 1 h (Supp.Fig. 3C). Foci induction was similar to that observed using fresh supernatant (Supp.Fig. 3B). These findings confirm that the decrease in foci induction after 24 h is not attributable to culture conditions.

**Fig. 4:**
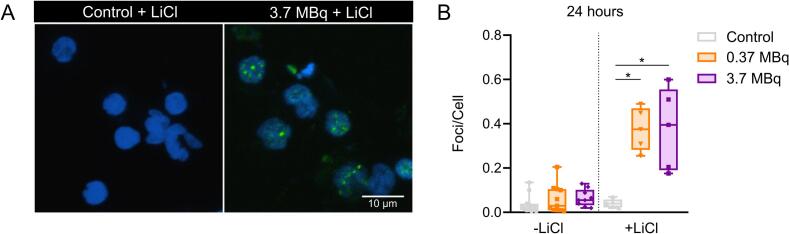
**Lithium Chloride Treatment Rescues γ-H2AX Foci Induction. A.** Representative image of γ-H2AX foci induced *ex vivo* in PBMCs from healthy individuals treated with 20 mM of lithium chloride (LiCl) under control conditions and exposed to ^131^I for 24 h. Indirect immunofluorescence for γ-H2AX (green) and nuclear counterstain with Hoechst 33,258 (blue). **B.***Ex vivo* measurement of γ-H2AX foci in PBMCs from healthy individuals treated with 20 mM of LiCl under control conditions and exposed to ^131^I (−LiCl, Kruskal-Wallis test, K-W = 3.170, n.s. Control, n = 12; 0.37 MBq, n = 11; 3.7 MBq, n = 9. + LiCl, Kruskal-Wallis test, K-W = 9.380, p = 0.0029, n = 5 for Control, 0.37 MBq and 3.7 MBq). Untreated cell cultures are plotted for comparison. (For interpretation of the references to colour in this figure legend, the reader is referred to the web version of this article.)

**Lithium Chloride Treatment Rescues γ-H2AX Foci Induction.** Based on our previous results, we hypothesized that the time-dependent decrease in radiation-induced foci could be attributed to changes in chromatin configuration. We treated 4 and 24-hour cell cultures with LiCl, a known chromatin-modifying agent. At 4 h LiCl treatment did not significantly alter γ-H2AX foci levels (Supp.Fig. 4). However, at 24 h LiCl reversed the observed decline in foci, restoring their levels to comparable values between the two activities (0.37 MBq: 0.376 ± 0.043, 3.7 MBq: 0.335 ± 0.063, Fig. 4). These findings confirm that LiCl can counteract the loss of detectable foci.

## Discussion

We investigated the DNA DSBs induced by continuous β^−^ particle irradiation at three different culture times and three distinct administered activity levels. To achieve a comprehensive assessment, we employed two complementary methodologies: dicentric chromosomes assay and γ-H2AX foci detection.

The results obtained from the cytogenetic assay DCA show that there is an increase in CAs in continuous exposure ^131^I that follows a linear trend. This highlights the difference between the radiobiological effects of continuous irradiation and acute X-ray exposures, with the latter exhibiting a quadratic-linear behaviour. The CA frequencies induced by ^131^I were less than those observed for equivalent X-ray doses. This difference can be attributed to a lower dose rate, which provides more time for the repair of sublethal lesions, enhancing the overall DNA repair capacity. The induction of CAs and micronuclei with X-rays or γ-rays at low dose rates shows a decrease in the quadratic coefficient (β) of the LQ model, reaching linearity [[29], [30], [31]]. Continuous exposure with β^−^ particles demonstrates that the data are a better fit for a strictly linear model. This behaviour is consistent with a previous report on blood samples exposed to another β^−^ emitter (^90^Y), at activities ranging from 40 to 400 kBq for 96 h [32]. Further studies with different activities and times, comparing different emitters (α, β, γ-rays) under continuous exposure conditions will be necessary to avoid underestimation of the dose absorbed in treatment with TRT.

Our results show an increase in the number of γ-H2AX foci in the first hours of exposure, in agreement with the kinetics reported in previous studies [[33], [34], [35]]. However, after 24 h of continuous incubation with the radionuclide, i.e. increasing the absorbed dose, γ-H2AX foci were no longer detected. The disappearance of foci could be attributed to the enhancement of DSB repair mechanisms after continuous exposure. However, under our protracted irradiation conditions, new DNA damage should be continuously occurring as seen in the DCA results. This suggests that the decrease in the number of foci detected at 24 h may not be representative of the induced damage and other factors (such as chromatin reconfiguration or changes of proteins involved in repair) could influence foci dynamics. This is supported by Lowe and collaborators (2020), who showed that chronic low-dose-rate γ-irradiation reduces histone protein levels despite ongoing DNA damage [36]. Moreover, Mariotti and collaborators (2013) described changes in the kinetics of foci formation after multiple fractions of *ex vivo* irradiation in primary human fibroblasts [34]. They postulated that H2AX phosphorylation may not be essential for activation of DNA damage response mechanisms once the system has been perturbed and the DNA damage repair machinery has already been activated. Changes in the kinetics of γ-H2AX foci formation in fibroblasts exposed to protracted irradiation have been related to chromatin condensation changes, particularly in the generation of heterochromatin foci associated to senescence (SAHF) [28,37]. Some studies have shown that ionizing radiation induces senescence in PBMCs, for instance, in patients following radiotherapy [38,39]. This senescent state is accompanied by epigenetic changes and chromatin remodeling [[40], [41], [42], [43], [44], [45]], as observed in senescent T cells, where a downregulation of H2AX expression has been specifically reported [46].

Several clinical studies have reported increased γ-H2AX foci in PBMCs from patients receiving ^131^I therapy. In these studies, foci peak within 4 h after administration and remain elevated for several days [[47], [48], [49]]. This discrepancy with our results highlights fundamental differences between exposure conditions. We irradiated uniformly and continuously all cells, which allowed us to perform a mechanistic analysis of DNA damage. Under *in vivo* irradiation conditions, factors such as blood circulation, heterogeneous dose distribution, and cell trafficking make accurate estimation of individual cell exposure difficult, contributing to the lack of a clear dose–response relationship. Our findings indicate that after 24 h of continuous exposure, γ-H2AX foci are no longer detectable, suggesting that *in vivo* foci counts may underestimate DNA damage in cells exposed to prolonged irradiation.

In summary, γ-H2AX is a useful marker for DNA double-strand damage, but its interpretation requires a thorough understanding of the continuous irradiation conditions and cellular mechanisms involved. Here, we show that continuous irradiation promotes DNA damage that could be underestimated when using the γ-H2AX assay. Adaptation of our experimental conditions with LiCl demonstrates that continuous exposure results in more damage than that observed under protracted exposure conditions. This constitutes a key consideration for adapting this assay if it is to be used to complement TRT imaging-based dosimetry. Further studies are needed to elucidate DNA damage dynamics and validate this biomarker for its use in TRT.

## Conclusion

Radiation-induced CAs and γ-H2AX foci were analyzed in blood cells after continuous β^−^ exposure at different activities and times. Here, we demonstrate that DNA damage induced by continuous exposure does not follow the LQ model. The CAs dose–response curve showed a linear behaviour, consistent with protracted irradiation at low dose rates, and revealed lower frequency of CAs than acute X-ray irradiation. A rapid increase in the number of γ-H2AX foci was observed after initial exposure, followed by their decrease at 24 h despite the ongoing irradiation from the radionuclide. LiCl treatment reversed the decrease in foci at this time, suggesting that chromatin configuration plays a crucial role in the induction of damage. This highlights the time-dependent dynamics of H2AX phosphorylation and its limitations as a marker in *in vivo* conditions, as it may underestimate DNA damage.

Although radiation-induced CAs quantification by DCA is a reliable method for estimating dose in continuous exposure, it is a labor intensive and time-consuming technique. Therefore, future research should focus on identifying a more rapid biomarker that faithfully reflects radiation-induced effects over time. This could be either adapting the γ-H2AX assay for *in vivo* applications, modeling its behaviour during protracted radiation exposure, or identifying a different radiation exposure biomarker that overcomes time limitation.

## Ethical approval

All the patient investigations conformed to the principles outlined in the Declaration of Helsinki and have been performed with the permission Res. # 4100 MS released by the responsible Provincial Commission of Ethics and Evaluation of Human Health Research Projects (CEEPISH), Ministry of Health of Río Negro, Argentina. Since peripheral blood from healthy adult volunteers was obtained by venipuncture, written informed consent was implemented. Additional privacy protection measures were taken. This article does not contain any studies with animals performed by any of the authors.

## Data availability statement

All data generated or analyzed during this study are included in this manuscript (and its supplementary information files).

## CRediT authorship contribution statement

**Laura Mazzitelli-Fuentes:** Conceptualization, Formal analysis, Investigation, Methodology, Validation, Visualization, Writing – original draft, Writing – review & editing. **Lara Negrin:** Conceptualization, Formal analysis, Investigation, Methodology, Validation, Visualization, Writing – original draft, Writing – review & editing. **Virginia Venier:** Investigation, Methodology, Resources. **Humberto Romano:** Investigation, Methodology, Resources. **Lucia Pereira:** Investigation, Methodology, Resources. **Jerónimo Leberle:** Investigation, Methodology, Resources. **Maria Soledad Ausas:** Investigation, Methodology, Resources. **Luisa V. Biolatti:** Conceptualization, Funding acquisition, Project administration, Resources, Supervision, Validation.

## Funding

This work was supported by INTECNUS Foundation and the National Atomic Energy Commission (CNEA), Argentina. Dr Luisa V. Biolatti and Professor Ananya Choudhury are both funded through the National Institute for Health and Care Research (NIHR) Manchester Biomedical Research Centre (BRC) (NIHR203308).

## Declaration of competing interest

The authors declare that they have no known competing financial interests or personal relationships that could have appeared to influence the work reported in this paper.
