# Supplementary material for: Continuous β− particle exposure: A study of DNA damage in ex vivo peripheral blood mononuclear cells irradiation with Radioiodine
Source: Clin Transl Radiat Oncol. 2025 Sep 1;55:101040. doi: 10.1016/j.ctro.2025.101040 (PMC12444485; doi:10.1016/j.ctro.2025.101040)
Supplement: Supplementary Data 1 [file mmc1.docx]

**Supplementary Material A**

**Methods and Materials**

**Blood Sampling, Cell Culture, and Irradiation.** Peripheral blood (PB) was obtained from healthy volunteers (4 men, 9 women; mean age ± SD: 36.9 ± 3.9) after written informed consent (Provincial Commission on Ethics and Evaluation of Research Projects in Human Health (CEEPISH), Ministry of Health of Rio Negro, Argentina (Resolution # 4100 MS). Blood collection and irradiation were performed at the Nuclear Medicine Service of INTECNUS Foundation; sample processing was carried out at the Radiobiology Laboratory (CNEA-INTECNUS). Blood samples from each donor was drawn into Li-heparin tubes, diluted 1:1 with RPMI-1640 medium (pH=7.2) supplemented with 10% Fetal Bovine Serum (FBS) and 1% Penicillin-Streptomycin (10.000 U/ml), and incubated with 0.37, 1.85 or 3.7 MBq of [¹³¹I]NaI solution (Radiofarma, Bacon) at 37°C for 1, 4 and 24 h. The activity of the [¹³¹I]NaI solution was measured using a dose calibrator and the volume administered was adjusted to the desired activity, never exceeding 250 µL per sample. Non-irradiated samples were used as controls. Additionally, for comparison with external-beam irradiation, PB samples from two healthy donors (1 man and 1 woman: 30 and 35 years-old, respectively) were irradiated with 15 MV X-rays at doses of 0.10, 0.25, 0.5, 1.0, 1.5, 2.0, 3.0 y 4.0 Gy using a clinical linear accelerator (Elekta Synergy^®^ Platform). After centrifugation (600 g, 10 min), the supernatant was discarded and pellet washed with RPMI-1640 and then suspended 1:1 in phosphate-buffered saline (PBS, pH=7.4). A cell viability assay was performed using the Live/Dead Cell Viability/Cytotoxicity Assay Kit (30002, Biotium, Fremont, CA) to confirm that there were no significant cytotoxic effects under the culture conditions. Similar viability was observed across all treatments (see Supplementary Material B, Supp. Fig. 1).

**Calculation of Absorbed Dose to Blood.** Absorbed doses were calculated using the MIRD method [^26^](https://www.zotero.org/google-docs/?WF5ofc). In our *ex vivo* model, blood is both the target and the source organ. The absorbed dose was determined by:

$D_{blood} =Ã S (r_{blood}\leftarrow r_{blood})$ (1)

where the cumulated activity (Ã), or integral of activity over time, was calculated as:

$Ã = \frac{A_{0}}{\lambda_{eff}}(1 - e^{-\lambda t})$ (2)

with *A_0_* being the initial activity, λ the physical decay constant of ¹³¹I (λ = 0.0036 h⁻¹, corresponding to T½ = 8.02 days). Since biological decay is absent, physical decay equals effective decay, $\lambda_{eff}=\lambda_{p}$.

The S factor was calculated as follows:

$S (r_{blood}\leftarrow r_{blood}) =\sum_{i} \frac{y_{i}E_{i}\phi_{i}}{m_{T}} ,$ (3)

where y_ᵢ_​ is the emission yield of each β⁻ disintegration (particles per decay), E_i​_ is the corresponding mean energy per transition (with a weighted average E_i_ = 181.85 keV), φ_ᵢ_ is the absorbed fraction (assumed to be = 1) and *m_T_* = 0.004 kg.

We assumed a homogeneous distribution of activity within 4 mL of blood (density 1 g/mL), and that all particles deposit their energy locally, due to their short range. Considering these assumptions and radiation data for ¹³¹I, the resulting S-factor was 7.28 10^-12^ Gy Bq^-1^ s^-1^.

**Cell Viability Assay.** After irradiation, PBMCs were isolated by Ficoll-Paque PLUS (Cytiva) gradient centrifugation according to the manufacturer’s instructions. PBMCs were diluted 1:3 in 2% FBS/PBS, centrifuged at 200g for 10 minutes, and the supernatant was discarded. This step was done twice, following a centrifugation of 500g. The remaining cells were stained using a cell viability assay, Live/Dead Cell Viability/Cytotoxicity Assay Kit (30002, Biotium, Fremont, CA), to confirm that there were no significant cytotoxic effects under the culture conditions. Cells were incubated with calcein (2 µM) and [ethidium](https://www.sciencedirect.com/topics/pharmacology-toxicology-and-pharmaceutical-science/ethidium) [homodimer](https://www.sciencedirect.com/topics/pharmacology-toxicology-and-pharmaceutical-science/homodimer) III (4 µM) for 30 min in dark at room temperature. Cells were transferred to a slide and mounted with PBS. Images were acquired by using a Confocal Fluorescence Microscope (LSM 980, Carl Zeiss).

**Dicentric Chromosome Assay.** Dicentric chromosome assay was performed using the standard method outlined in the IAEA’s Cytogenetic Dosimetry Guideline. Briefly, after irradiation, 500 µl of blood were cultured with RPMI-1640 medium (pH=7.2) supplemented with 10% FBS, 1% Penicillin-Streptomycin (10.000 U/ml), 2% Phytohemagglutinin (Sartorius, 223800912) and 1% Bromodeoxyuridine (Sigma, HMBF2060V) in 15 ml centrifuge tubes. Cells were cultured at 37 °C in a humidified 5% CO_2_ atmosphere for 48 h. Colcemid was added 2 h prior to the completion of the culture to arrest lymphocytes at metaphase (0.2 µg/ml, Biological Industries, 2213303). Subsequently, the cells were treated with hypotonic 0.075M KCl solution, and the resulting pellet was fixed and washed in a 1:3 Carnoy's fixative. Chromosome spreads were then prepared on slides. Finally, samples were subjected to Fluorescence plus Giemsa staining. Metaphases were acquired and processed using the CytoVision® Image Analysis and Capture System (Leica Microsystems). The frequency of dicentrics and centric rings chromosomes was manually scored in a minimum of 600 metaphases per treatment. For the dose-response curves of chromosome aberrations (both dicentrics and rings), the iterative weighted least squares method was used, adjusting the observed values to the linear or linear-quadratic model (Y = c + 𝛼D + 𝛽D^2^) using the software Dose Estimate_v5.3 software.

**Immunofluorescence and Image Acquisition.** DSBs were detected using the γ-H2AX foci assay. After irradiation, PBMCs were isolated by Ficoll-Paque PLUS (Cytiva) gradient centrifugation according to the manufacturer’s instructions. PBMCs were diluted 1:3 in 2% FBS/PBS, centrifuged at 200 g for 10 min, and the supernatant was discarded. This step was done twice, following a centrifugation of 500 g. PBMCs cells were fixed in 4% PFA for 15 min at room temperature (RT), washed with PBS and stored at 4°C. γ-H2AX foci were detected using indirect immunofluorescence. Cells were blocked with 5% FBS + 0.3% TritonX-100 in PBS for 1 h and incubated overnight at 4°C with primary antibodies diluted in PBS. Primary antibodies used were: rabbit polyclonal anti-Phospho-Histone H2A.X (Ser139) 1:800 (2577, Cell Signal). PBMCs were washed with 0.3% TritonX-100/PBS for 5 minutes, and incubated with secondary antibodies coupled to Alexa Fluor 488 (1:1000 dilution in PBS) for 2 h at RT. Cells were then washed in 0.3% TritonX-100/PBS for 5 minutes, and stained with Hoescht 33258 during 15 minutes at RT. Cells were washed in 0.3% TritonX-100/PBS, transferred to a slide and mounted with glycerol. Images were acquired by using a Confocal Fluorescence Microscope (LSM 980, Carl Zeiss), acquiring stacks at 1 μm intervals to cover all focal planes. Foci quantification was performed with FIJI software (ImageJ v1.53). A total of 200 cells were scored per treatment, except in a few cases (10 out of 156) where methodological limitations prevented obtaining a large number of cells, in which case all detectable cells were counted, ranging from 32 to 194 cells.

**Lithium Chloride Treatment.** To investigate whether foci induction is influenced by chromatin state, we conducted an experiment using LiCl, a compound which has been proposed to relax heterochromatin foci, and rescue the delayed γ-H2AX foci formation associated with cellular senescence [^28,29^](https://www.zotero.org/google-docs/?KFZcok). Building on this rationale, LiCl (3019741, Thermofisher) was added to control or 3.7 MBq cell cultures at a final concentration of 20 mM for 24 h at 37°C, according to Oizumi et al. 2024[^28^](https://www.zotero.org/google-docs/?JLIy8n). The procedures described previously were followed.

**Statistical Analysis.** Data were analyzed using GraphPad Prism version 8.0.1 (GraphPad Software, CA, USA). Every data set was tested by the Grubb’s outlier test, with alpha = 0.05. Normality was assessed using the Shapiro-Wilk test. Homoscedasticity was analyzed by Bartlett's test. In scatter plots, data points are shown as mean ± SEM. Box plots display the distribution of the data, with the box representing the interquartile range (IQR), line indicates median, and whiskers extending to the minimum and maximum values. Statistical significance was assumed when p < 0.05. For multiple comparisons, Dunn’s test was applied. Statistical significance is indicated as follows: *p < 0.05; **p < 0.01; ***p < 0.001; ****p < 0.0001.
