# Supplementary material for: Continuous β− particle exposure: A study of DNA damage in ex vivo peripheral blood mononuclear cells irradiation with Radioiodine
Source: Clin Transl Radiat Oncol. 2025 Sep 1;55:101040. doi: 10.1016/j.ctro.2025.101040 (PMC12444485; doi:10.1016/j.ctro.2025.101040)
Supplement: Supplementary Data 2 [file mmc2.docx]

**Supplementary Material B**

**Supplementary Figures**
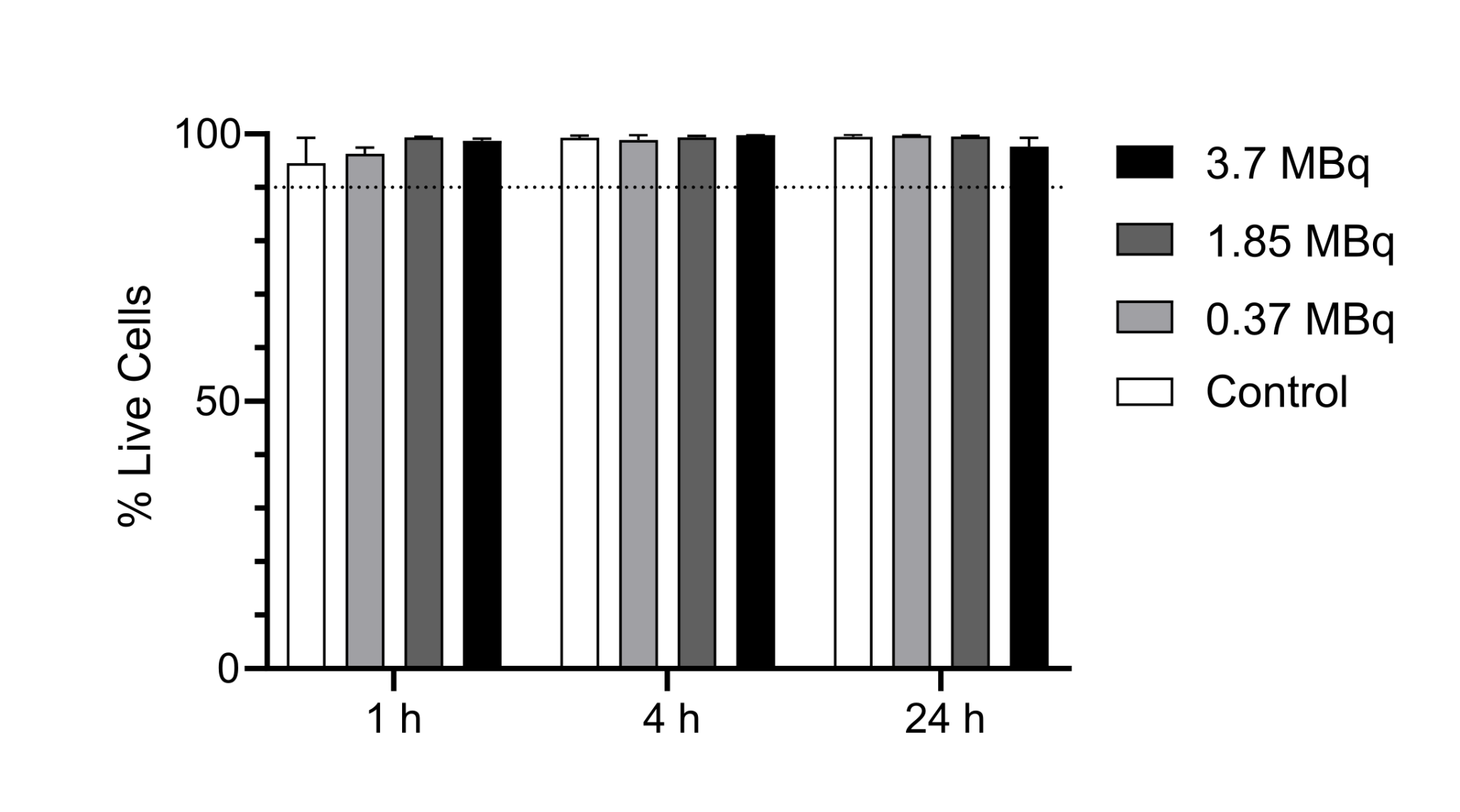


**Supplementary Figure 1.** Percentage of Live cells (calcein^+^ cells. Biotium. Fremont. CA) for each activity administered and time period. Data are presented as mean ± SEM. n=3. Dashed line depicts 90% of cell viability.

**
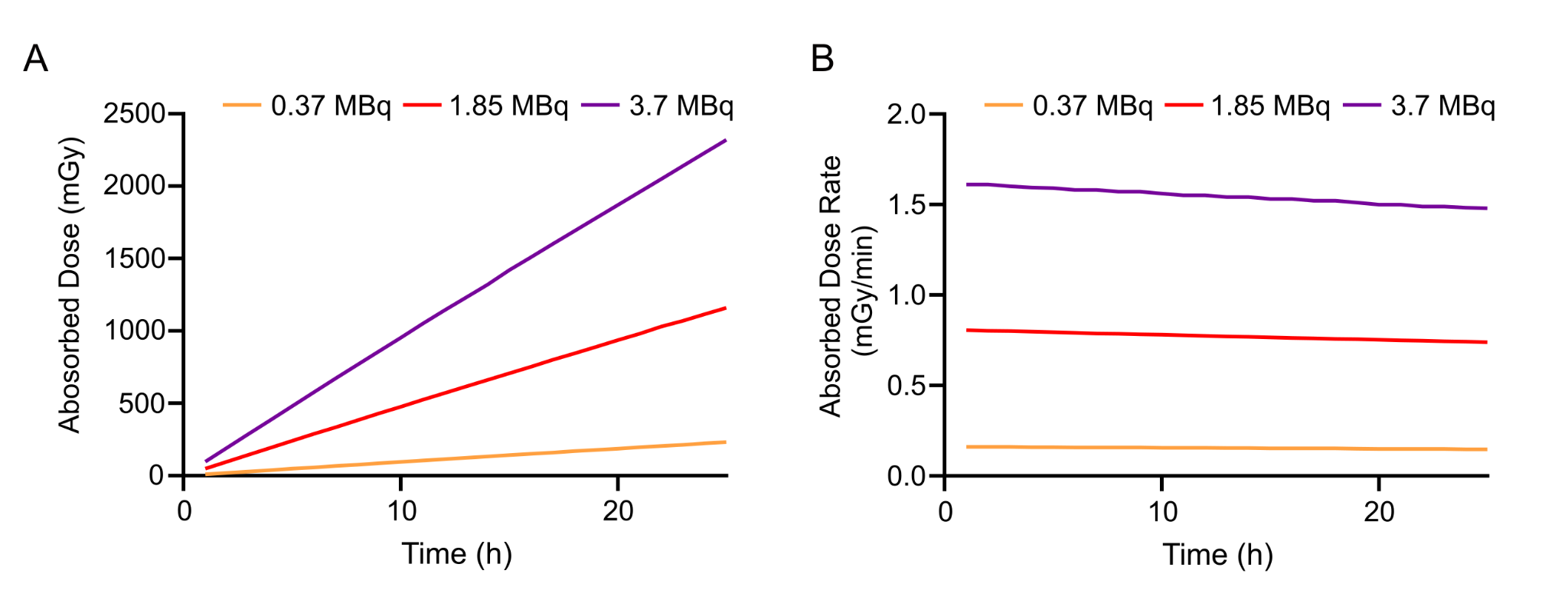
**

**Supplementary Figure 2. Absorbed Dose and Dose Rate in Peripheral Blood for Each Administered Activity.** Absorbed dose (A) and dose rate (B) in peripheral blood for each administered activity calculated by MIRD method.


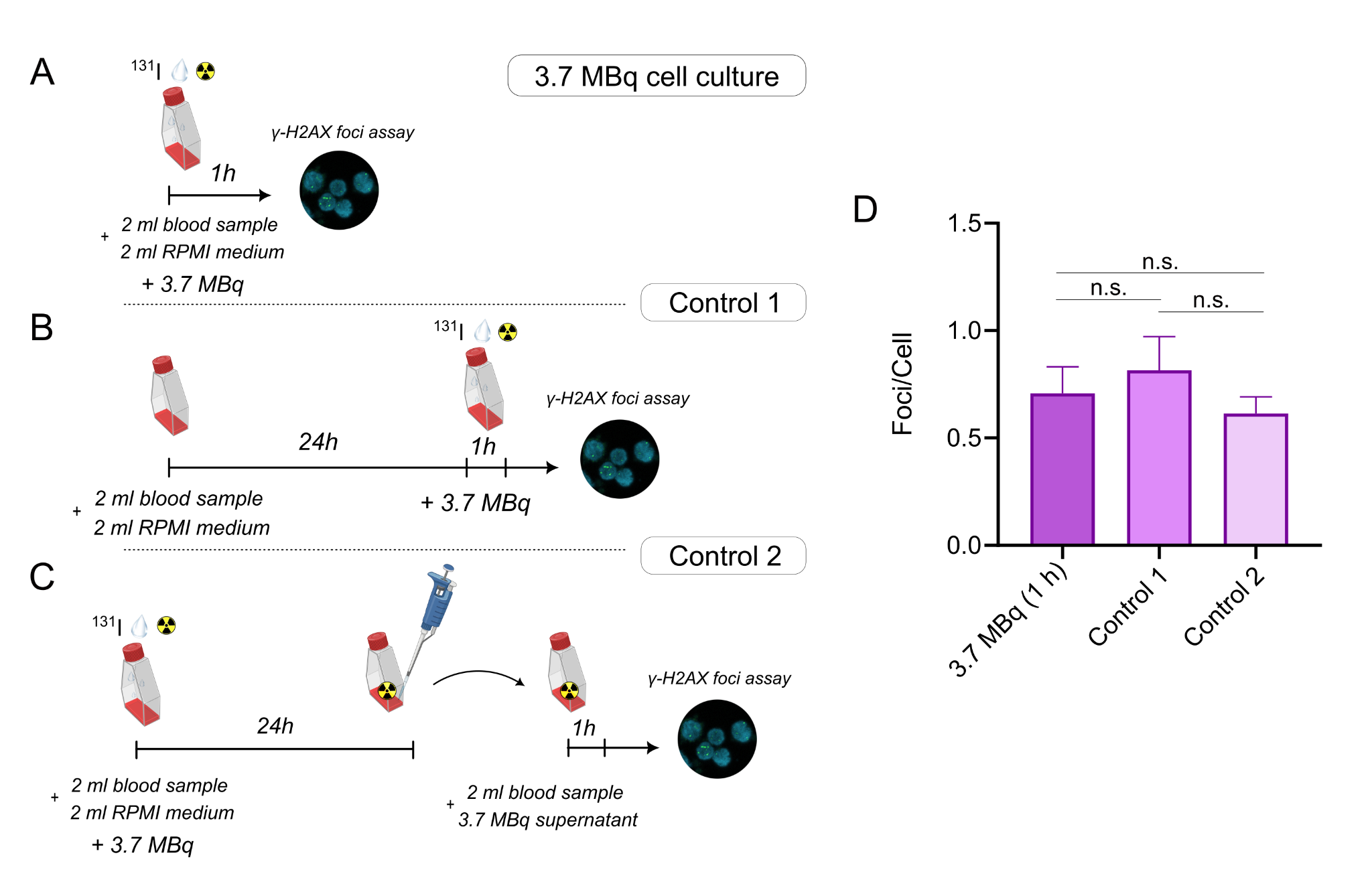


**Supplementary Figure 3. Control of Culture Conditions. A.-C** Experimental designs (created with BioRender.com). **A.**  PB cells were cultured for 24 h with 3.7 MBq of ^131^I. **B.** PB cultures were maintained for 24 h and subsequently irradiated 1 h with 3.7 MBq of ^131^I. **C.** PB cells were cultured for 1 h with the supernatant from a 24-h culture exposed to 3.7 MBq of ^131^I. **D.** *Ex vivo* measurement of γ-H2AX foci in PBMCs from healthy individuals exposed to ^131^I (Kruskal-Wallis test. K-W=0.8107. n.s. 3.7 MBq (1 h). n=11; Control 1. n=4; Control 2. n=3). PBMCs. peripheral blood mononuclear cells; PB. peripheral blood.

**
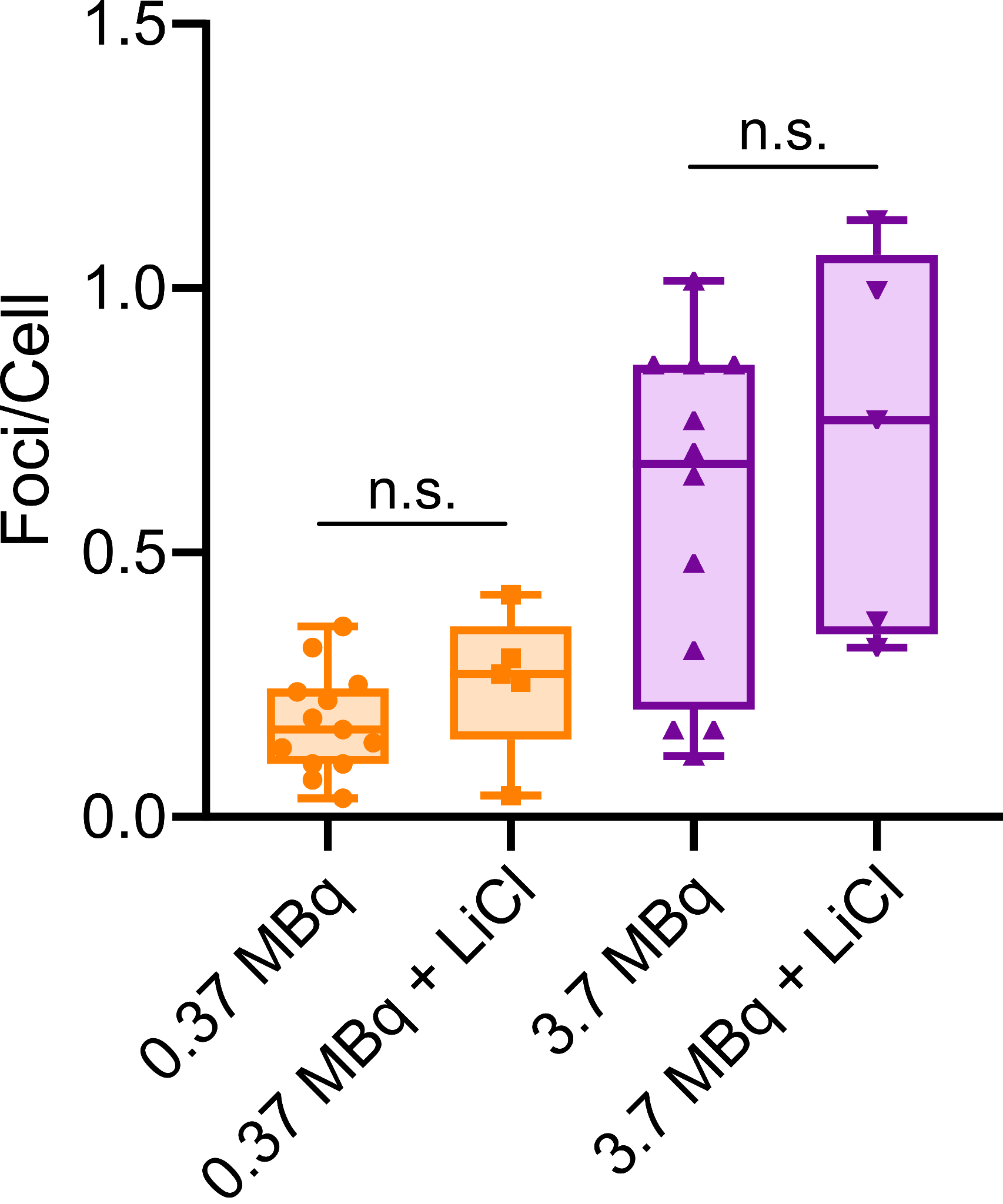
**

**Supplementary Figure 4. Lithium Chloride Treatment. A.** *Ex vivo* measurement of γ-H2AX foci in PBMCs from healthy individuals treated with 20 mM of LiCl in 4-hour cultures exposed to 0.37 and 3.7 MBq of ^131^I (n=5). Untreated cell cultures are plotted for comparison (n=13). (Kruskal-Wallis test. K-W=15.46, p=0.0015. Dunn's multiple comparisons test, 0.37 MBq vs. 0.37 MBq + LiCl, n.s., 3.7 MBq vs. 3.7 MBq + LiCl, n.s.).

**Supplementary Table 1.** Parameters of the linear fits corresponding to the dose-response curves presented in Figure 3E.

|  | **1 hour** | **4 hours** | **24 hours** |
| --- | --- | --- | --- |
| **Best-fit values** |  |  |  |
| Slope | 0.006234 | 0.001224 | 3.528e-005 |
| Y-intercept | 0.1348 | 0.1036 | 0.04040 |
| X-intercept | -21.63 | -84.67 | -1145 |
| **Goodness of Fit** |  |  |  |
| R square | 0.4399 | 0.4703 | 0.1365 |
| **Equation** | Y = 0.006234*X + 0.1348 | Y = 0.001224*X + 0.1036 | Y = 3.528e-005*X + 0.04040 |

**Supplementary Table 2.** Parameters of the exponential fits corresponding to the dose-response curves presented in Figure 3F.

|  | **0.37 MBq** | **1.85 MBq** | **3.7 MBq** |
| --- | --- | --- | --- |
| **Best-fit values** |  |  |  |
| Y0 | 0.2118 | 0.5188 | 0.7711 |
| K | 0.04223 | 0.09275 | 0.07688 |
| **Goodness of Fit** |  |  |  |
| R squared | 0,3138 | 0,3464 | 0,3750 |
| **Equation** | Y(t) = 0.2118* e^(–0.04223*t) | Y(t) = 0.5188 * e^(–0.09275·t) | Y(t) = 0.7711*e^(–0.07688·t) |
